# Carpal tunnel syndrome in paediatric patients: A novel association with Kosaki overgrowth syndrome

**DOI:** 10.1016/j.jpra.2020.07.001

**Published:** 2020-07-27

**Authors:** Harriet Walker, Alison Foster, Trevor Cole, Andrea Jester

**Affiliations:** a Hand and Upper Limb Service, Plastic and Reconstructive Surgery, Birmingham Women's and Children's NHS Foundation Trust, Birmingham, UK; b West Midlands Regional Genetics Service and Birmingham Health Partners, Birmingham Women's and Children's Hospitals NHS Foundation Trust, Birmingham, UK

**Keywords:** Kosaki overgrowth syndrome, KOGS, Carpal tunnel syndrome, Paediatric carpal tunnel syndrome

## Abstract

Carpal tunnel syndrome in a paediatric population is vanishingly rare and usually associated with lysosomal storage disorders such as mucopolysaccharidosis (MPS). Overgrowth syndromes similarly are rare and are characterised by increased skeletal growth alongside typical dysmorphic features and intellectual delay and as such the acronym OGID (overgrowth intellectual delay) is now widely used. Kosaki overgrowth syndrome (KOGS) is a newly recognised OGID with only 6 cases to date reported in the literature. Here we report a 7th case of KOGS with a new finding of carpal tunnel syndrome not previously described. We discuss similarities between the intraoperative findings during carpal tunnel decompression with findings seen in patients with MPS.

## Background

Overgrowth syndromes are a heterogenic group of disorders characterised by non-hormonally accelerated skeletal growth presenting from the fetal to early childhood period and are frequently associated with typical dysmorphic features and varying degree of developmental delay.^1^^,^^2^ The term OGID encapsulates the two key components of overgrowth and intellectual delay.^1^ Many OGID syndromes are caused by abnormal regulation of signalling cascades controlling cell growth and differentiation, most commonly due to perturbations of epigenetic regulation of gene expression.^1^^,^^2^ Kosaki overgrowth syndrome (KOGS) is a recently described overgrowth with only 6 cases reported to date.^3^^,^^4^ Characteristic features include scoliosis, overgrowth, hyperelastic skin, neurological deterioration and typical facies.^5^ Mutations in platelet derived growth factor receptor-B (PDGFRB) with knock-on abnormalities in the cell-surface tyrosine kinase receptor PDGFRβ found on mesenchymally derived cells are responsible for the clinical features.^6^ Other conditions associated with mutations in this gene include Penttinen syndrome, infantile myofibromatosis and myeloproliferative disorder with eosinophilia, although KOGS is the only disorder associated predominantly with overgrowth.^3^ Two genetic variants have been identified within the same juxtamembrane domain of PDGFRB: c.1751>*G* p.(Pro584Arg) and c.1696T>*C* p.(Trp566Arg) with no clear differences in the pattern of clinical manifestations.^3^^,^^5^^,^^7^ Here we describe a diagnosis of KOGS in a 7-year-old boy with severe carpal tunnel syndrome, a feature not previously described. We discuss specific intraoperative findings in comparison to other paediatric causes of carpal tunnel syndrome.

## Case presentation

The patient was born at 38 weeks weighing 3.7 kg. Concerns were raised at aged 7 months over abnormal cranial growth and a referral to a national craniosynostosis service was made. Imaging revealed multiple suture synostosis with premature fusion of metopic, sagittal and unicoronal sutures and cysts in the thalamus, basal ganglia and cerebral peduncle. An acute hydrocephalus was treated by VP shunt insertion which required 5 revisions. Development was delayed although language remains normal and he began to walk at 14 months. Vision is impaired secondary to hydrocephalus and hearing is reduced bilaterally from severe glue ear requiring the use of hearing aids.

Dysmorphic features include brachycephaly, sloping forehead, thickened supraorbital ridges, hypertelorism, proptosis, downslanting palpebral fissures, wide nasal bridge and tip, malar flattening, midface retrusion, smooth philtrum, thin vermillion of upper lip, everted vermillion of lower lip and prominent ears with forward facing and uplifted earlobes. Delayed eruption of secondary dentition resulted in wide spaced teeth.

There is a dry and hyperelastic quality to the skin. At aged 7 there is no scoliosis however the patient exhibits a broad chest and pectus excavatum. There is overgrowth within all 4 limbs with widened metatarsals and metacarpals and evidence of dysostosis multiplex. His hands and feet are broad with stubby fingers. There are progressive flexion contractures of all digits, more marked at the distal interphalangeal joints. Both thumbs demonstrate triggering with a palpable Notta's node at the metacarpophalangeal joints. Nerve conductions studies were performed due to the “typical” appearance of digital flexion contracture, similar to those seen in Mucopolysaccharidosis and nocturnal waking. These showed severe bilateral carpal tunnel syndrome, worse on the left.

The patient was recruited to the Phenotyping of Overgrowth Disorders (POD) study (CPMS 19,361) and underwent testing on a custom designed next-generation sequencing panel of 44 overgrowth genes. A known pathogenic variant c.1751C>*G* p.(Pro584Arg) was identified in PDGFRB. De novo status was confirmed on Sanger sequencing of the proband and both parents by the West Midlands Regional Genetics Laboratory (WMRGL).

**Figure 1:**
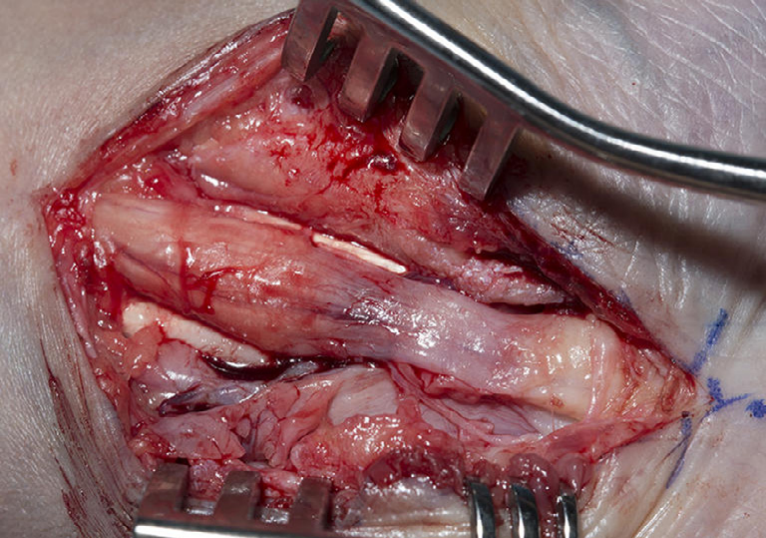
Median nerve at decompression, note the bulbous morphology proximally with distal thinning.

**Figure 2:**
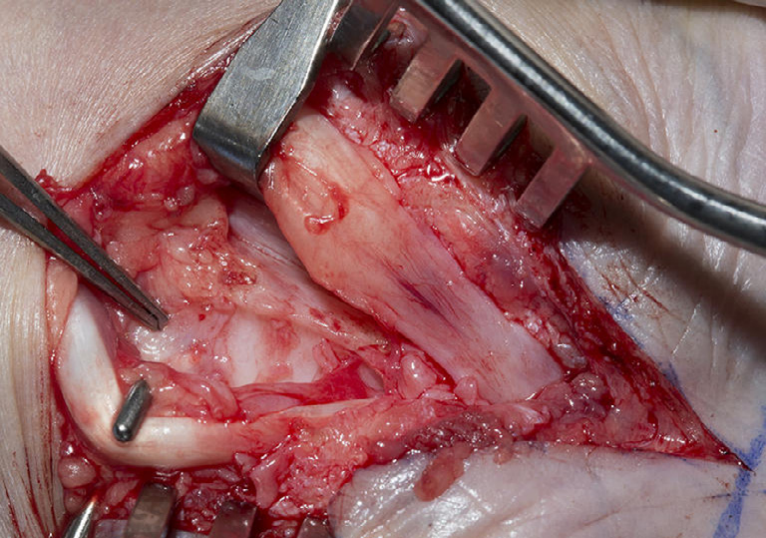
Extensive scarring of the flexor tendons after retraction of the median nerve.

Bilateral carpal tunnel decompression was performed under general anaesthesia with forearm block, tourniquet control and loupe magnification. The incision extended from the palm into the distal forearm visualising the full length of the retinaculum flexorum and exposing median nerve and all flexor tendons. The flexor retinaculum was macroscopically normal however there was visible thickening of the carpal complex. The median nerve was seen to be bulbous proximally and thinned beneath the retinaculum (Figure 1). There was widespread scar and subsynovial connective tissue infiltrating the flexor tendons, especially in the deep compartment (Figure 2). A tenosynovectomy was performed which led to immediate improvement in the passive range of movement of the interphalangeal joints. Histopathological examination of the scar tissue revealed extensive fibrotic tissue. The connective tissue was heavily collagenised with moderate numbers of non-atypical fibroblasts and no vacuolated cells. The incision was closed in layers with an absorbable monofilament to the dermis and a braided absorbable suture to the skin. The wound was dressed with paraffin impregnated tulle, gauze and crepe bandage.

Post-operatively the patient experienced an uncomplicated recovery and at 3 months nocturnal waking had improved, pain had diminished and active and passive range of movement of fingers had improved. Ongoing follow-up continues in a multi-disciplinary fashion with clinical genetics, hand surgery, orthopaedic surgery, occupational therapy, audiology and ophthalmology.

## Discussion

Kosaki overgrowth syndrome is a recently recognised OGID syndrome with only 5 female and 1 male patient reported to date. A recent article by Gawlinski in 2017 described 58 symptoms associated with KOGS and proposed a core 18 features common to all reported patients.^3^ The case we have presented here exhibits 16 of these (Table 1). The features seen in KOGS have a tendency to be progressive and further phenotypic overlap with previous cases may evolve.

**Table 1:** 18 core features of KOGS reproduced from Gawlinski et al. with comparison to presented patient.

Common feature of KOGS described in all patients in literature (both genotypes)	Present in current patient
Overgrowth	+
Tall stature	+
Hypertelorism	+
Downslanted palpebral fissures	+
Wide nasal bridge	+
Wide nasal base	+
Pointed chin	–
Hyperelastic skin	+
Thin, fragile skin	+
Prominent forehead and supraorbital ridge	+
Broad nasal tip	+
Large hands	+
Malar flattening	+
Cupid-bow shaped upper lip	–
Thin upper lip	+
Proptosis	+
Smooth philtrum	+
Periventricular white matter lesions	+

Our patient is the first to date who has developed bilateral median nerve compression, progressive flexion contracture of fingers and triggering of thumbs, although it is interesting to note that Pond et al. in 2018 report a patient with carpal tunnel syndrome in the context of overgrowth and a PDGFRB mutation, reported as distinct from KOGS but showing significant phenotypical overlap.^6^ Other conditions associated with mutations in this gene include Penttinen syndrome, idiopathic basal ganglia calcification type 4, infantile myofibromatosis and myeloproliferative disorder with eosinophilia although KOGS and the patient of Pond et al. are the only cases associated with overgrowth.^3^

Carpal tunnel syndrome in a paediatric population is extremely uncommon. Nearly all cases are seen in the context of the lysosomal storage disorders mucopolysaccharidosis (MPS) or mucolipidosis. Here median nerve compression is due to glycosaminoglycan deposition in the subsynovial connective tissue leading to compression of the median nerve against the scarred flexor tendons. This necessitates partial median nerve epineurectomy and tenosynovectomy of flexor tendons as well as simple decompression.^8^ Other rare causes include diabetes mellitus, obesity, a hereditary neuropathy with liability to pressure palsies, trauma^9^ and macrodactyly. Unlike in an adult, young children often struggle to localise or describe their symptoms so diagnosis is usually late and based on clinical signs such as thenar wasting, clumsiness, digital gnawing and difficulty with fine tasks. This is especially true in patients with developmental delay.^10^ In the case reported here, the finding of extensive fibrous tissue surrounding the flexor tendons is more in keeping with the pathophysiology of median nerve compression in MPS 1,2 or 6 rather than being secondary to somatic overgrowth. This finding may have implications on the underlying pathology in KOGS, although further work will need to be done to confirm this.

In this article we have reported a 7th patient with Kosaki overgrowth syndrome. The presence of carpal tunnel syndrome is a new and potentially important finding which may shed light on the underlying pathophysiology of this disorder.

## Declaration of Competing Interest

No authors have any conflict of interest to declare in relation to this submitted article.
